# Methamphetamine-Induced Dopamine-Independent Alterations in Striatal Gene Expression in the 6-Hydroxydopamine Hemiparkinsonian Rats

**DOI:** 10.1371/journal.pone.0015643

**Published:** 2010-12-13

**Authors:** Jean Lud Cadet, Christie Brannock, Irina N. Krasnova, Bruce Ladenheim, Michael T. McCoy, Jenny Chou, Elin Lehrmann, William H. Wood, Kevin G. Becker, Yun Wang

**Affiliations:** 1 Molecular Neuropsychiatry Research Branch, Intramural Research Program, National Institute on Drug Abuse/National Institutes of Health/Department of Health and Human Services, Baltimore, Maryland, United States of America; 2 Gene Expression and Genomics Unit, Intramural Research Program, National Institute on Drug Abuse/National Institutes of Health/Department of Health and Human Services, Baltimore, Maryland, United States of America; The Mental Health Research Institute of Victoria, Australia

## Abstract

Unilateral injections of 6-hydroxydopamine into the medial forebrain bundle are used extensively as a model of Parkinson's disease. The present experiments sought to identify genes that were affected in the dopamine (DA)–denervated striatum after 6-hydroxydopamine-induced destruction of the nigrostriatal dopaminergic pathway in the rat. We also examined whether a single injection of methamphetamine (METH) (2.5 mg/kg) known to cause changes in gene expression in the normally DA-innervated striatum could still influence striatal gene expression in the absence of DA. Unilateral injections of 6-hydroxydopamine into the medial forebrain bundle resulted in METH-induced rotational behaviors ipsilateral to the lesioned side and total striatal DA depletion on the lesioned side. This injection also caused decrease in striatal serotonin (5-HT) and 5-hydroxyindoleacetic acid (5-HIAA) levels. DA depletion was associated with increases in 5-HIAA/5-HT ratios that were potentiated by the METH injection. Microarray analyses revealed changes (± 1.7-fold, p<0.025) in the expression of 67 genes on the lesioned side in comparison to the intact side of the saline-treated hemiparkinsonian animals. These include follistatin, neuromedin U, and tachykinin 2 which were up-regulated. METH administration caused increases in the expression of *c-fos, Egr1,* and *Nor-1* on the intact side. On the DA-depleted side, METH administration also increased the expression of 61 genes including *Pdgf-d* and *Cox-2*. There were METH-induced changes in 16 genes that were common in the DA-innervated and DA-depleted sides. These include *c-fos* and *Nor-1* which show greater changes on the normal DA side. Thus, the present study documents, for the first time, that METH mediated DA-independent changes in the levels of transcripts of several genes in the DA-denervated striatum. Our results also implicate 5-HT as a potential player in these METH-induced alterations in gene expression because the METH injection also caused significant increases in 5-HIAA/5-HT ratios on the DA-depleted side.

## Introduction

Dysfunctions of basal ganglionic structures are the substrates for Huntington's and Parkinson's diseases [1], [2]. Rats that received unilateral injections of 6-hydroxydopamine (6-OHDA) in the nigrostriatal dopaminergic system are used as a model for Parkinson's disease. These animals exhibit ipsilateral rotations after administration of indirect dopamine (DA) agonists and contralateral rotations after direct DA agonists [3]–[8]. These behaviors are related to unilateral changes in the expression of striatal dopaminergic markers [3]–[5], [9]–[12]. In addition, striatal DA depletion is associated with changes in the expression of mRNA precursors for some neuropeptides including enkephalin, substance P, and dynorphin [13]–[15]. Moreover, injections of direct DA receptor agonists that stimulate postsynaptic DA receptors cause substantial changes in the expression of several genes in the DA-depleted striatum [16]–[20]. However, it is not clear to what extent indirect agonists, such as the amphetamines that release DA and other neurotransmitters [21]–[25] might also influence gene expression in the DA-depleted striatum.

Methamphetamine (METH) is an indirect agonist that induces release of DA and serotonin (5-HT) in the brain [21], [22], [24]–[26]. Repeated injections of large METH doses also cause delayed increases in glutamate release in the striatum [23], [27], [28]. In addition, METH administration influences striatal gene expression in animals with normal dopaminergic innervation [29]–[33]. The METH-induced transcriptional changes depend on stimulation of DA and glutamate receptors [29], [31], [33]. However, the extent to which METH might also induce changes in gene expression in the absence of DA innervation has not been clarified. Studies that have examined the effects of indirect agonists on striatal gene expression in hemiparkinsonian rodents have measured the expression of only a few genes or proteins. For example, repeated injections of amphetamine (5 mg/kg) to rats with unilateral nigral 6-OHDA lesions caused increased striatal dynorphin-like immunoreactivity on the intact but not on the lesioned side [34]. Neither did amphetamine administration induce any changes in met-enkephalin expression on the lesioned side [34]. In contrast, Chritin et al. [35] reported that amphetamine caused up-regulated Fos protein expression on both sides of hemiparkinsonian rats, with the increases being of smaller magnitude on the DA-denervated side.

Given the importance of DA in the mediation of striatal synaptic plasticity and striatum-dependent behaviors, we thought it is likely that DA depletion might be associated with changes in the expression of a larger number of genes than those described so far in intrinsic striatal cells [13]–[15]. The possibility also existed that indirect DA agonists including METH, which causes release of other neurotransmitters such as 5-HT [22], might affect the expression of some genes in a DA-independent fashion. Therefore, the present study was undertaken to analyze global gene expression in the DA-denervated striatum and to quantify striatal METH-induced transcriptional responses after 6-OHDA-induced lesions of the nigrostriatal dopaminergic pathway. Our results show that there are other genes that are affected in the DA-depleted striatum in addition to those previously reported [13]–[15]. We also found that METH administration does indeed cause changes in the expression of several genes in the DA-depleted striatum. Our observations further suggest that METH-induced increased 5-HT turnover might, in part, be responsible for the later changes.

## Methods

### Animals

Male Sprague-Dawley rats (Charles Rivers Laboratories, Raleigh, NC), weighing 270–300 g at the beginning of the experiments were used in the present study. Animals were housed in a humidity- and temperature-controlled room and were given free access to food and water. All animal procedures were performed according to the National Institutes of Health *Guide for the Care and Use of Laboratory Animals* and were approved by the Animal Care and Use Committee of the National Institute on Drug Abuse, Intramural Research Program. The research was conducted under Animal Study Protocol #09-CNRB-25.

### 6-OHDA lesioning

Unilateral lesions were performed under anesthesia with chloral hydrate (400 mg/kg, i.p.). After immobilization on a stereotaxic frame (model 940; David Kopf Instruments), a hole was drilled in the skull for injections of 6-OHDA in the medial forebrain bundle (MFB). 6-OHDA (2 µg/µl ×5 µl in 0.9% NaCl containing 0.2 mg/ml ascorbic acid) was unilaterally injected into the left MFB (−4.4 mm AP, 1.2 mm ML relative to bregma and 8.4 mm below skull) over 4 minutes. At the end of each injection, the micropipette was left in place for an additional 5 min and then withdrawn slowly to prevent reflux of the solution.

### Rotation

Rotational behavior was evaluated using a multichannel rotometer system (RotoMax, AccuScan Instruments, Inc). Contraversive or ipsiversive rotational behaviors were induced by subcutaneous injection of apomorphine (APO) (0.05 mg/kg) on day 24 or (±) METH HCl (2.5 mg/kg, s.c) on day 31, respectively, after the 6-OHDA injection. Each animal was placed in a cylindrical test chamber for 90 min. The highest consecutive clockwise and counter-clockwise rotations over 60 min were used for analysis.

### METH treatment and tissue collection

One week after measuring METH-induced rotation, the animals were divided into two groups based on rotational behavior. The two matched groups of animals were injected with either saline or METH (2.5 mg/kg, i.p.) and then euthanized 2 hours after the injection. Additional control animals that did not get 6-OHDA injections were also used. Their brains were quickly removed, tissues were dissected on ice, snap frozen on dry ice, and stored at −80°C until used in HPLC, microarray, and quantitative PCR (qPCR) experiments. The experimental groups were: saline-treated controls (SC), METH-treated controls (MC), non-lesioned side of saline-treated 6-OHDA-injected rats (SNL), lesioned side of saline-treated 6-OHDA-injected rats (SL), non-lesioned side of METH-treated 6-OHDA-injected animals (MNL), and lesioned side of METH-treated 6-OHDA-injected animals (ML).

### HPLC

For monoamine analysis, the brain regions were homogenized in 0.01 M HClO_4_ and centrifuged at 14, 000× g for 15 min. DA, 3,4-dihydroxyphenylacetic acid (DOPAC), homovanillic acid (HVA), 5-HT and 5-hydroxyindoleacetic acid (5-HIAA) levels were analyzed in the brain tissue extracts using HPLC with electrochemical detector [36], [37].

### RNA extraction, microarray hybridization, and data analysis

Total RNA was isolated using RNeasy Midi kit (Qiagen, Valencia, CA). RNA integrity was assessed using an Agilent 2100 Bioanalyzer (Agilent, Palo Alto, CA) and showed no degradation. Microarray hybridization was carried out using RatRef-12 Expression BeadChips arrays (22, 523 probes) (Illumina Inc., San Diego, CA). In brief, a 600 ng aliquot of total RNA from each striatal sample was amplified using Illumina RNA Amplification kit (Ambion, Austin, TX). Single-stranded RNA (cRNA) was generated and labeled by incorporating biotin-16-UTP (Roche Diagnostics, Indianapolis, IN). 750 ng of each cRNA sample were hybridized to Illumina arrays at 55°C overnight according to the Whole-Genome Gene Expression Protocol for BeadStation (Illumina Inc.). Hybridized biotinylated cRNA was detected with Cyanine3-streptavidin (GE Healthcare, Piscataway, NJ) and quantified using Illumina's BeadStation 500GX Genetic Analysis Systems scanner.

The microarray data reported in the manuscript are in accordance with MIAME guidelines. The raw data for the analyses have been deposited in the NCBI GEO database, series record GSE24233 (http://www.ncbi.nlm.nih.gov/geo/query/acc.cgi?acc=GSE24233). The Illumina BeadStudio software was used to measure fluorescent hybridization signals. Data were extracted by BeadStudio (Illumina) and analyzed using GeneSpring software v. 7.3.1 (Silicon Genetics, Redwood City, CA). Raw data were imported into GeneSpring and normalized using global normalization. The normalized data were used to identify changes in gene expression in the 4 group comparisons: SL vs SNL, ML vs SNL, MNL vs SNL, and ML vs SL. A gene was identified as significantly changed if it showed increased or decreased expression according to an arbitrary cut-off of 1.7-fold changes at p<0.025, according to GeneSpring statistical package (unpaired t-test). In previous studies, genes identified by similar criteria were consistently validated by qPCR [31], [38], [39].

### qPCR

Total RNA extracted from the striatum was used to confirm the expression of genes of interest by qRT-PCR. In brief, unpooled total RNA obtained from 8–12 striata per group was reverse-transcribed with oligo dT primers and RT for PCR kit (Clontech, Palo Alto, CA). PCR experiments were done using the Chroma4 RT-PCR Detection System and iQ SYBR Green Supermix (BioRad, Hercules, CA). Sequences for gene-specific primers corresponding to PCR targets were obtained using LightCycler Probe Design software (Roche). The primers were synthesized and HPLC-purified at the Synthesis and Sequencing Facility of Johns Hopkins University (Baltimore, MD). The primers are listed in Table S1. qPCR values were normalized using light chain of clathrin and quantified. The results are reported as relative changes calculated as the ratios of normalized gene expression data of each group compared to the control group injected with saline (SC).

### Statistical Analysis

Statistical analysis for the HPLC and PCR data was performed using analysis of variance (ANOVA) followed by Fisher's protected least significant difference post-hoc comparison (StatView 4.02, SAS Institute, Cary, NC). Values are shown as means ± SEM. The null hypothesis was rejected at p<0.05.

## Results

### Functional characterization of the unilateral 6-OHDA nigrostriatal lesion

#### Rotation

**Figure 1:**
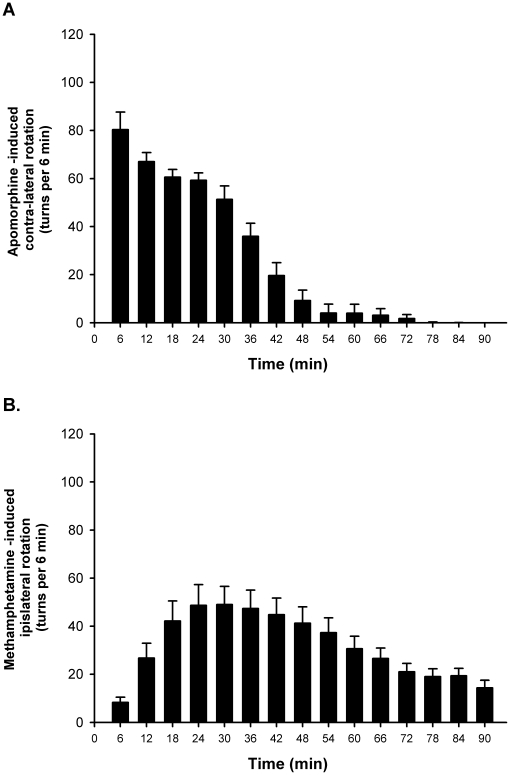
Apomorphine- and methamphetamine-induced rotations in 6-OHDA hemiparkinsonian rats. The animals were injected with 6-OHDA as described in the text. Rotation was measured after injection of apomorphine (APO) (0.05 mg/kg) (A) or methamphetamine (METH) (2.5 mg/kg) (B).

We assessed the effectiveness of the 6-OHDA lesions by both behavioral and biochemical means. Figure 1 shows the effects of APO and METH on contraversive and ipsiversive rotational behaviors, respectively. We found time-dependent changes in APO-induced turns, which peaked within the first 6 min after injection of the drug (Figure 1A). APO-induced contralateral turns were stable for the first 30 min and then rapidly tapered to being nonexistent by 54 min after drug. METH-induced ipsiversive turns became apparent within the first 12 min after the drug injection and peaked at around 30 min. METH-induced rotations remained prominent for about 1 hour and were still measurable at 90 min after METH injection (Figure 1B).

#### Biochemistry

We found no differences in the levels of either DA or its metabolites, DOPAC and HVA on the intact side of hemiparkinsonian rats in comparison to control animals (Table 1). Unilateral injections of 6-OHDA in the MFB caused complete loss of DA, DOPAC, and HVA (Table 1). Injection of METH prior to euthanizing the animals did not affect DA, DOPAC, and HVA levels. METH did not cause any significant changes in DOPAC/DA or HVA/DA ratios in any of the groups (data not shown). Table 1 also shows the effects of unilateral 6-OHDA lesions on 5-HT and 5-HIAA levels as well as 5-HIAA/5-HT ratios in the striatum. We found significant decreases in 5-HT concentrations on the side of the 6-OHDA injections in the saline- (−37%) and in METH-treated (−51%) rats. 5-HIAA levels were also affected on the lesioned side of the saline- (−26.5%) and METH-treated (−26.4%) animals. In addition, injections of METH caused significant increases in 5-HIAA levels in the control group (+33%) and on the intact side of the hemiparkinsonian rats (+24%). We also found increases in 5-HIAA/5-HT ratios on the 6-OHDA-lesioned side of the saline- (+24.4%) and METH-treated animals (+54.7%). The increases in 5-HIAA/5-HT ratios on the DA-depleted side of animals treated with METH were significantly higher than the ratios in all other groups. These findings are consistent with those of other investigators who have reported increases in 5-HIAA/5-HT ratios in animals that had suffered greater than 90% loss of striatal DA [40]. However, the potentiating effects of METH on 5-HIAA/5-HT ratios in the lesioned striatum have not been reported before.

**Table 1:** Effects of 6-OHDA and METH on striatal monoamine levels.

Monoamine	Control rats		Hemiparkinsonian rats			
	Saline	METH	Saline		METH	
	SC	MC	SNL (Contra)	SL (Ipsi)	MNL (Conta)	ML (Ipsi)
DA	100.0±7.9	95.5±13.6	114.9±12.4^b^	0.0±0.0^a^	110.0±12.8^b^	0.0±0.0^a^
DOPAC	100.0±13.6	84.8±12.2	116.0±10.3^b^	0.3±0.3^a^	91.1±13.8^b^	0.0±0.0^a^
HVA	100.0±8.8	113.4±14.9	111.7±10.7^b^	0.0±0.0^a^	113.2±11.5^b^	0.0±0.0^a^
5-HT	100.0±8.0	123.6±13.9	95.3±6.0^b^	62.6±11.5^a^	115.2±12.4^b^	49.1±7.4^a^
5-HIAA	100.0±4.0	133.3±9.2^a^	108.4±3.0^b^	73.4±8.0^a^	124.0±9.3a,b	73.6±7.7^a^
5-HIAA/5-HT	100.0±5.9	109.5±6.9^c^	111.8±5.6^c^	124.4±10.3a,c	107.0±5.4^b^	154.7±11.5^a^

The values represent means ± SEM (percentage changes with respect to the SC group) of 8–10 measurements per group. The groups are as described in the text. Statistical analyses were done by ANOVA followed by PLSD. Key to statistics:

a p<0.05 in comparison to the SC group;

b p<0.05, in comparison to the ipsilateral (Ipsi) side of similarly treated groups;

c p<0.05 in comparison to the 5-HIAA/5-HT ratio in the ML group.

### Dopamine depletion and METH-induced changes in mRNA levels in the striatum

#### Microarray analysis

**Figure 2:**
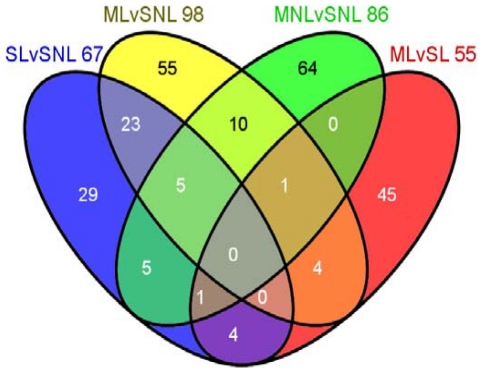
Differential profiles of transcript levels in saline- and METH-treated 6-OHDA-induced hemiparkinsonian rats. The Venn diagram shows the distinctiveness and the overlap of genes identified by the four separate comparisons shown in the figure. The rats were treated and euthanized as described in the text. RNA was extracted from striatal tissues obtained from both sides of the brains of saline- and METH-injected hemiparkinsonian rats. Microarray analyses were performed as described in the text. Genes were identified as differently expressed if they showed greater than ±1.7-fold changes at p<0.025.

In order to provide a panoramic view of METH effects on gene expression in the DA-depleted striatum, we used large scale microarray analysis with rat Illumina arrays that contain about 23, 000 genes. Figure 2 shows a Venn diagram of the comparisons between 4 groups of interest: SL vs SNL, ML vs SNL, MNL vs SNL, and ML vs SL. The identity of the affected genes is given in Tables 2, 3, 4, and 5 which list the full names of the genes. The Venn diagram shows 67 genes that were affected in the DA-denervated side in comparison to the intact side (SL vs SNL). Of these, 45 were up-regulated whereas 22 were down-regulated (Table 2). Up-regulated genes include *Ldhc, Stab2, Nmu, Fst, Nts,* and *Tac2* whereas down-regulated transcripts included *Tac1* (Table 2). METH injection caused changes in the expression of 86 genes in the intact side (MNL) in comparison to the SNL side (Table 3). Genes of interest that were up-regulated include transcription factors *c-fos, Junb, Egr2,* and *Nr4a3 (Nor-1)*. METH administration caused alterations in mRNA levels for 98 genes on the lesioned side in comparison to the SNL side (ML vs SNL) (Table 4). These include 61 up-regulated and 37 down-regulated genes, with only 16 of these genes being also affected in the MNL vs SNL comparison (Tables 3 and 4). Of the 98 genes affected in the ML vs SNL group, 28 were also found in the SL vs SNL comparison, with their expression, for the most part, not being further affected by the METH administration (Tables 2 and 4). Genes of interest found only in the ML vs SNL comparison were *Syt10* and *Cox-2,* which were up-regulated by the METH injection. One of the common genes between the two sets of comparisons (MNL vs SNL and ML vs SNL) is *Nr4a3 (Nor-1)* which shows higher expression in the MNL vs SNL group. When we evaluated gene expression in the ML versus SL group, another set of 55 genes was identified, with 30 being up-regulated and 25 being down-regulated (Table 5). As shown in the Venn diagram, only 5 of these transcripts were also found in the ML vs SNL group. These include *c-fos* and *Hcst* (Tables 4 and 5). We also compared gene expression between the MNL and ML groups and found that several transcription factors including *c-fos, Egr1, Egr2,* and *Junb* were significantly up-regulated in the MNL group (data not shown, see Table 6 for qPCR validation).

**Table 2:** Partial list of genes showing changes in the DA-depleted striatum of hemiparkinsonian rats.

(SL/SNL)		
Fold Changes	Gene Symbol	Description
22.47	Gucy1b2	guanylate cyclase 1, soluble, beta 2
21.85	Igsf7	immunoglobulin superfamily, member 7
16.31	Tyms	thymidylate synthase
13.28	Ldhc	lactate dehydrogenase C
8.63	Stab2	stabilin 2
8.44	Nav2	neuron navigator 2
8.42	Mcpt4	mast cell protease 4
8.13	Bcl9l_predicted	B-cell CLL/lymphoma 9-like
6.41	Nmu	neuromedin U
5.62	Msln	mesothelin
3.86	Pcsk1	proprotein convertase subtilisin/kexin type 1
3.49	Fst	follistatin
3.08	Upb1	ureidopropionase, beta
2.99	Nts_predicted	neurotensin
2.84	Tac2	tachykinin 2
2.71	Plek2_predicted	pleckstrin 2
2.59	Pctp	phosphatidylcholine transfer protein
2.50	Slc26a2	solute carrier family 26 (sulfate transporter), member 2
2.48	Pkp2	plakophilin 2
2.40	Olr35	olfactory receptor 35
2.19	E2f1	E2F transcription factor 1
1.96	Il3ra	interleukin 3 receptor, alpha chain
1.89	Inhba	inhibin beta-A
1.85	Lama5	laminin, alpha 5
1.83	Kif22	kinesin family member 22
1.80	Amhr2	anti-Mullerian hormone type 2 receptor
1.74	Sox11	SRY-box containing gene 11
1.72	Cflar	CASP8 and FADD-like apoptosis regulator
1.71	Fxyd5	FXYD domain-containing ion transport regulator 5
−1.91	Tac1	tachykinin 1
−2.07	Plekhg2_predicted	pleckstrin homology domain containing, family G member 2
−2.89	Cd44	CD44 antigen
−3.08	P2ry1	purinergic receptor P2Y, G-protein coupled 1
−5.06	Il1r2	interleukin 1 receptor, type II
−6.47	Sox5	SRY-box containing gene 5
−7.60	Kcnmb3_predicted	potassium large conductance calcium-activated channel, M beta 3
−12.77	Hoxa1	homeo box A1

This partial list of genes was generated from the SL vs SNL comparison. To be included the genes had to meet the inclusion criteria: ±1.7-fold at p<0.025. The genes are listed in descending order according to fold changes.

**Table 3:** Partial list of METH-induced changes in the levels of gene transcripts on the intact striatum of hemiparkinsonian rats.

(MNL/SNL)		
Fold Changes	Gene Symbol	Description
29.82	Gucy1b2	guanylate cyclase 1, soluble, beta 2
16.61	Kcnf1	potassium voltage-gated channel, subfamily F, member 1
15.06	Stxbp5l_predicted	syntaxin binding protein 5-like
14.05	Gtpbp1_predicted	GTP binding protein 1
11.12	Lrrc21	leucine rich repeat containing 21
8.11	Stab2	stabilin 2
5.99	Ntf3	neurotrophin 3
5.16	c-fos	FBJ murine osteosarcoma viral oncogene homolog
4.68	Egr4	early growth response 4
4.54	Egr2	early growth response 2
4.53	Nr4a3	nuclear receptor subfamily 4, group A, member 3
3.36	Pcsk1	proprotein convertase subtilisin/kexin type 1
2.66	Junb	Jun-B oncogene
2.58	Klf10	Kruppel-like factor 10
2.44	Phlda1	pleckstrin homology-like domain, family A, member 1
2.42	Rasl11a	RAS-like family 11 member A
2.37	Fcgr3	Fc receptor, IgG, low affinity III
2.23	Egr1	early growth response 1
2.06	Ppap2a	phosphatidic acid phosphatase 2a
2.05	Mrgpre	MAS-related GPR, member E
1.95	Vgf	VGF nerve growth factor inducible
1.93	Rasa2	RAS p21 protein activator 2
1.90	Dusp1	dual specificity phosphatase 1
1.83	Dnajb5_predicted	DnaJ (Hsp40) homolog, subfamily B, member 5
1.81	Nts_predicted	neurotensin
1.80	Pgap1	GPI deacylase
1.77	Rem2	rad and gem related GTP binding protein 2
1.73	Rgs2	regulator of G-protein signaling 2
1.71	Snf1lk	SNF1-like kinase
−1.85	Nox4	NADPH oxidase 4
−2.21	V1rk1	vomeronasal 1 receptor, k1
−2.49	Nxt2_predicted	nuclear transport factor 2-like export factor 2
−3.14	Sulf1	sulfatase 1
−7.84	Chrm5	cholinergic receptor, muscarinic 5
−10.88	Syt2	synaptotagmin II
−11.72	Rnf43_predicted	ring finger protein 43
−17.88	Ocil	osteoclast inhibitory lectin
−18.31	Pak4_predicted	p21 (CDKN1A)-activated kinase 4
−19.25	Krt10	keratin 10

The data were generated from the MNL vs SNL comparison. The genes are listed in descending order according to fold changes in comparison to the SNL group.

**Table 4:** Partial list of genes showing METH-induced changes in the DA-depleted striatum.

(ML/SNL)		
Fold Changes	Gene Symbol	Description
14.20	Ung_predicted	uracil-DNA glycosylase
13.08	Kcnf1	potassium voltage-gated channel, subfamily F, member 1
10.21	Mrgprb5	MAS-related G protein-coupled receptor, member B5
8.82	Stab2	stabilin 2
8.11	Stxbp5l_predicted	syntaxin binding protein 5-like
6.70	Nmu	neuromedin U
6.09	Eln	elastin
4.85	Pcsk1	proprotein convertase subtilisin/kexin type 1
3.90	Nts_predicted	neurotensin
3.24	Upb1	ureidopropionase, beta
3.12	Fst	follistatin
3.03	c-fos	FBJ murine osteosarcoma viral oncogene homolog
2.99	Plk1	polo-like kinase 1 (Drosophila)
2.87	Inhba	inhibin beta-A
2.84	Tac2	tachykinin 2
2.79	Ptgs2	prostaglandin-endoperoxide synthase 2
2.29	Phex	phosphate regulating gene homologus to endopeptidases on X chrom
2.23	Nr4a3	nuclear receptor subfamily 4, group A, member 3
2.19	Apbb1ip	amyloid beta precursor protein-binding, B1 interacting protein
2.18	Cflar	CASP8 and FADD-like apoptosis regulator
2.11	Syt10	synaptotagmin X
1.96	Klf10	Kruppel-like factor 10
1.93	Kif22	kinesin family member 22
1.90	Syk	spleen tyrosine kinase
1.87	Egr4	early growth response 4
1.83	Mmp9	matrix metallopeptidase 9
1.73	Penk1	proenkephalin 1
1.72	E2f1	E2F transcription factor 1
−1.71	Tac1	tachykinin 1
−1.73	Top1	topoisomerase (DNA) I
−1.80	Il1r2	interleukin 1 receptor, type II
−1.97	Opn4	opsin 4 (melanopsin)
−2.20	V1rk1	vomeronasal 1 receptor, k1
−2.39	Art5	ADP-ribosyltransferase 5
−2.44	Aox1	aldehyde oxidase 1
−2.63	Dfna5h	deafness, autosomal dominant 5 homolog (human)
−10.37	Prm1	protamine 1
−12.17	Krt10	keratin 10; type I keratin KA10
−16.05	Hcst	hematopoietic cell signal transducer

The partial list was generated from the ML vs SNL comparison. Inclusion criteria are as described in Table 2. The genes are listed in descending order according to fold changes in comparison to expression in the SNL group.

**Table 5:** METH-induced changes in levels of gene transcripts on the lesioned side of hemiparkinsonian rats.

(ML/SL)		
Fold Changes	Gene Symbol	Description
17.49	Olr784	olfactory receptor 784
14.27	Olr597	olfactory receptor 597
13.75	Papd4	PAP associated domain containing 4
11.86	Kirrel2	kin of IRRE like 2 (Drosophila)
11.69	Pdgfd	platelet-derived growth factor, D polypeptide
11.08	Espn	espin
10.37	V1rc1	vomeronasal V1r-type receptor V1rc1
9.72	Cd34	CD34 antigen
8.64	Olr1616	olfactory receptor 1616
7.14	Olr1332	olfactory receptor 1332
6.80	Psd2	pleckstrin and Sec7 domain containing 2
3.96	Fut9	fucosyltransferase 9
3.46	Wnt9b	wingless related MMTV integration site 9B
2.17	Cd44	CD44 antigen
2.02	Ptpns1l3	protein tyrosine phosphatase, non-receptor type substrate 1-like 3
1.97	Mmachc	methylmalonic aciduria cblC type, with homocystinuria
1.87	Synj2bp	synaptojanin 2 binding protein
1.82	c-fos	FBJ murine osteosarcoma viral oncogene homolog
−1.88	Daam2	dishevelled associated activator of morphogenesis 2
−2.07	Igfbp6	insulin-like growth factor binding protein 6
−2.14	Abcg2	ATP-binding cassette, sub-family G (WHITE), member 2
−7.70	Olr630	olfactory receptor 630
−9.16	Zfp697	zinc finger protein 697
−10.50	Msh5	mutS homolog 5 (E. coli)
−11.62	Cubn	cubilin (intrinsic factor-cobalamin receptor)
−12.03	Taar7e	trace-amine-associated receptor 7e
−12.80	V1rc6	vomeronasal V1r-type receptor V1rc6
−14.24	Arl12	ADP-ribosylation factor-like 12
−29.82	Hcst	hematopoietic cell signal transducer

The data were generated from the ML vs SL comparison. The genes are listed in descending order according to fold changes in comparison to expression in the SL group.

**Table 6:** Effects of METH on striatal expression of transcription factors in hemiparkinsonian rats.

Transcription factor	Control rats		Hemiparkinsonian rats			
	Saline	METH	Saline		METH	
	SC	MC	SNL	SL	MNL	ML
c-fos	1.00±0.11	3.26±0.35a,b	0.80±0.19^c^	1.09±0.17^c^	2.74±0.34a,b	1.75±0.24a,d
fos-b	1.00±0.13	2.96±0.43a,b	0.66±0.07^c^	0.86±0.12^c^	3.59±0.59a,b	1.59±0.26^d^
Fra1	1.00±0.12	0.95±0.05	1.20±0.17	0.97±0.17	0.83±0.07	0.98±0.11
Fra2	1.00±0.13	4.56±1.09a,b	0.75±0.09^c^	0.84±0.10^c^	4.19±0.73a,b	1.68±0.25
c-jun	1.00±0.12	1.32±0.14	1.33±0.25	1.29±0.16	1.24±0.17	1.45±0.21
Junb	1.00±0.19	1.70±0.17a,b	0.65±0.12^c^	0.59±0.07	1.58±0.19a,b	1.03±0.15
Jund	1.00±0.12	1.04±0.13b,c	1.37±0.16^c^	1.43±0.16^c^	2.24±0.20^a^	1.88±0.34^a^
Egr1	1.00±0.17	2.09±0.25a,b	0.78±0.08^c^	0.77±0.11^c^	2.08±0.23a,b	0.84±0.12
Egr2	1.00±0.18	3.82±0.60a,b,c	0.61±0.08^c^	0.60±0.09^c^	6.05±1.14a,b	1.08±0.20
Egr3	1.00±0.14	1.61±0.22a,c	0.88±0.08^c^	0.72±0.09^c^	2.18±0.32a,b	1.18±0.18
Nr4a1	1.00±0.20	4.60±0.58a,b	0.68±0.09^c^	2.09±0.30c,d	5.01±0.81a,b	2.26±0.31^d^
Nr4a2	1.00±0.27	1.60±0.51	1.32±0.24	1.59±0.32	1.24±0.36	1.28±0.50
Nr4a3	1.00±0.16	5.30±0.71a,b	1.10±0.19^c^	1.26±0.08^c^	6.28±0.72a,b	2.96±0.87a,d

The values represent means ± SEM (fold changes with respect to the SC group) of 8–10 measurements per group. The groups are as described in the text. Statistical analyses were done by ANOVA followed by PLSD. Key to statistics:

a p<0.05, in comparison to the SC group;

b p<0.05, in comparison to the ML group;

c p<0.05, in comparison to the MNL group;

d p<0.05 in comparison to the SNL group.

#### qPCR

**Figure 3:**
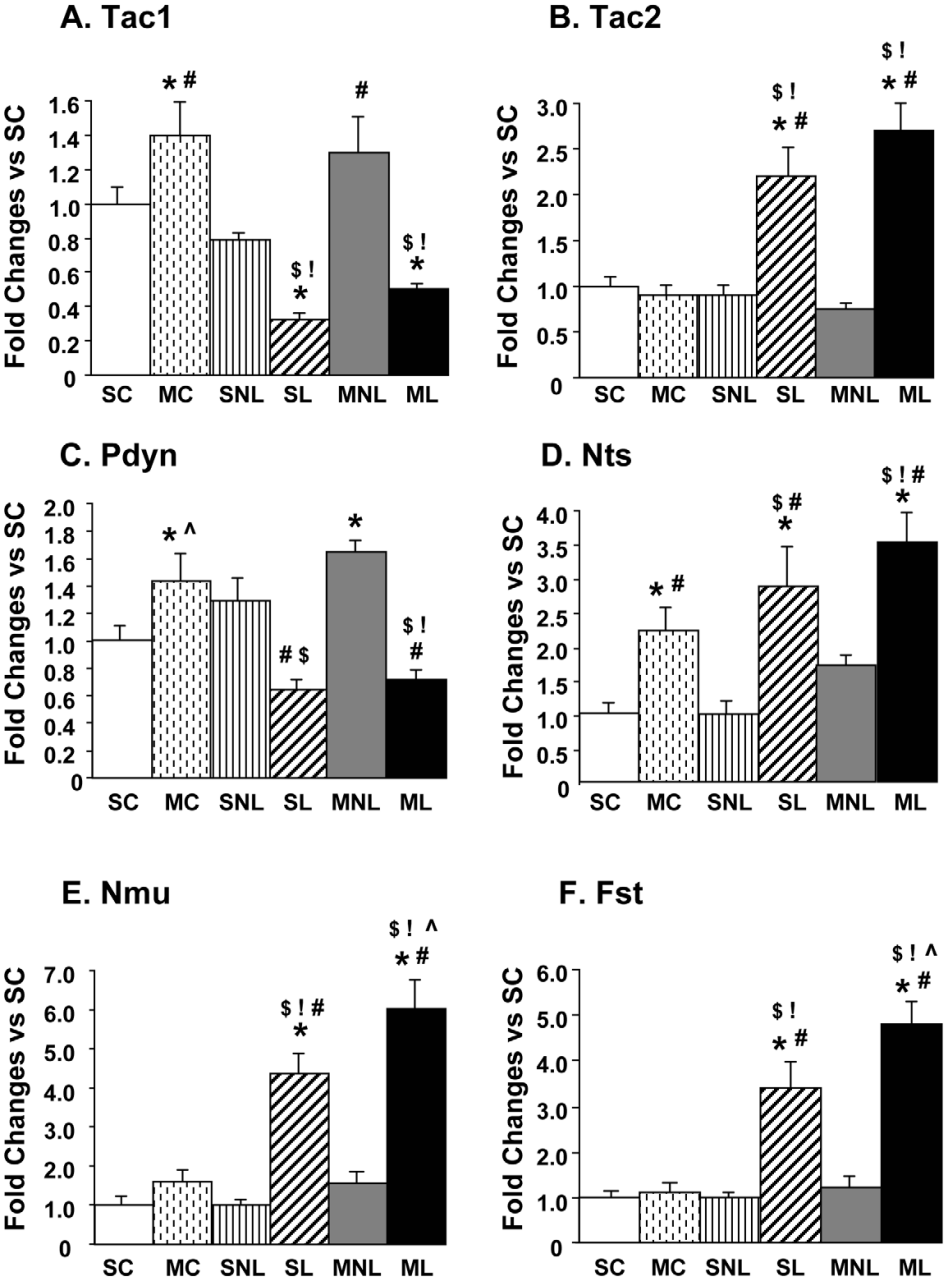
Effects of DA depletion and METH on striatal expression of *Tac1, Tac2, Pdyn, Nts, Nmu* and *Fst*. The values represent means ± SEM (fold changes in comparison to the SC group) of 6–10 measurements. Statistical analyses were done by ANOVA followed by PLSD. Key to statistics: Key to statistics: *p<0.05, in comparison to the SC group; #p<0.05, in comparison to the SNL group; !p<0.05, in comparison to the MC group; 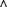
p<0.05, in comparison to the SL group; $p<0.05, in comparison to the MNL group.

Figure 3 shows the results of the qPCR validation for some of the genes affected on the lesioned side of the hemiparkinsonian rats treated with saline (SL vs SNL comparison). Consistent with the array data, DA depletion was associated with significant decreases (−68% of SC group) in *Tac1* (also called tachykinin 1, preprotachykinin A, substance P) expression on the DA depleted side (SL). The injection of METH did not significantly influence *Tac1* expression on the lesioned side (−50% of SC group). In contrast, the single METH injection caused small increases in normal animals (1.4-fold) and on the intact side (1.3-fold) of the 6-OHDA-treated rats (Figure 3A). In contrast to the effects on *Tac1,* DA depletion resulted in significant increases (2.2-fold) in the expression of *Tac2* (neurokinin B) (Figure 3B). METH administration did not induce any changes in *Tac2* expression in the control animals nor in the intact side of 6-OHDA lesioned animals. METH did not potentiate the effects of 6-OHDA on *Tac2* expression on the lesioned side (compare SL to ML groups in Figure 3B). There were also significant 6-OHDA-induced decreases in *Pdyn* expression in the SL group (Figure 3C). These 6-OHDA-induced changes were not affected by METH. In contrast, METH injection caused increases in *Pdyn* mRNA levels in the control animals and in the intact side of 6-OHDA-injected rats. Striatal DA depletion also induced significant increases (2.86-fold) in *Nts* mRNA levels (Figure 3D). The METH injection caused increases (2.21-fold) in *Nts* mRNA in the control animals (MC group), confirming previous results, which showed that METH induced a dramatic increase in striatal *Nts* mRNA levels [41]. METH injection did not cause further increases in *Nts* expression on the side of the lesion when compared to the saline-treated 6-OHDA-injected animals (compare SL to ML group). Nevertheless, the data for the ML group were significantly higher than those measured for the MC group (Figure 3D). There were also significant DA depletion-induced increases (4.36-fold) in *Nmu* mRNA levels in the 6-OHDA-treated animals (Figure 3E). METH injection did not change *Nmu* expression in the control animals (MC group) nor on the intact side of the lesioned animals. However, METH treatment enhanced the increases (6.04-fold) in *Nmu* expression on the DA- depleted side (compare SL to ML group). The qPCR experiment also replicated the array data for *Fst* expression, which showed significant increases (3.39-fold) on the DA-depleted side of hemiparkinsonian rats (Figure 3F). The METH-treated animals also showed increases (4.78-fold) in the lesioned side, with these changes being significantly higher than those observed in the saline-treated 6-OHDA-lesioned animals. The observations that the small METH dose caused further increases in *Fst* mRNA levels on the lesioned side of 6-OHDA-treated rats suggest that these METH-induced alterations in *Fst* expression might be independent of METH-induced DA release.

Table 6 shows the qPCR validation of the microarray data which identified multiple transcription factors affected on the intact side of the 6-OHDA-treated rats after METH injection (see Table 3). The effects of METH on the expression of immediate-early genes in the normally DA innervated striatum have been the subjects of several reports ([31] and references therein). However, because these studies were conducted in animals with normal neurotransmitter contents, conclusions drawn from these investigations might not help to fully characterize the role of DA and other neurotransmitters in these transcriptional responses. Therefore, for the purpose of thoroughness, we performed qPCR on other genes that fall within similar classes of transcription factors and that had been identified as affected by injections of higher METH doses [30], [31]. Except for *Fra1, c-jun,* and *Nr4a2 (Nurr1),* METH injection caused significant increases in the expression of the other members of these classes of transcription factors. We found that METH caused increases in c-fos mRNA levels on both the intact (2.7-fold) and on the DA-denervated (1.7-fold) side (Table 6). Another gene of interest is *Jund*. METH injection caused similar increases in *Jund* expression on both the intact (2.2-fold) and lesioned (1.9-fold) striata of the hemiparkinsonian rats (Table 6). We found significant increases in the expression of *Nr4a1 (Nur77)* in the denervated striatum in comparison to the intact side (SL vs SNL) of hemiparkinsonian rats. These data are different from the findings of microarray analysis that did not identify *Nr4a1* as being changed in the SL group. These might be related to the somewhat stringent criteria used in the array analysis and/or to the greater sensitivity of qPCR. The values in the SL group were also comparable to those measured on the lesioned side after METH (ML group) administration, implying that METH did not influence *Nr4a1* expression in the absence of DA.

Other transcription factors of interest are the EGRs [42]. *Egr2* expression was substantially more induced by METH injection in the intact side (6.1-fold) of the hemiparkinsonian rats in comparison to control animals (3.8-fold) (Table 6). Similar differential responses were observed for *Egr3* expression after METH administration to the control and 6-OHDA-injected animals. This is in contrast to the effects of METH on *Egr1* which were almost identical in the control and lesioned rats.

**Figure 4:**
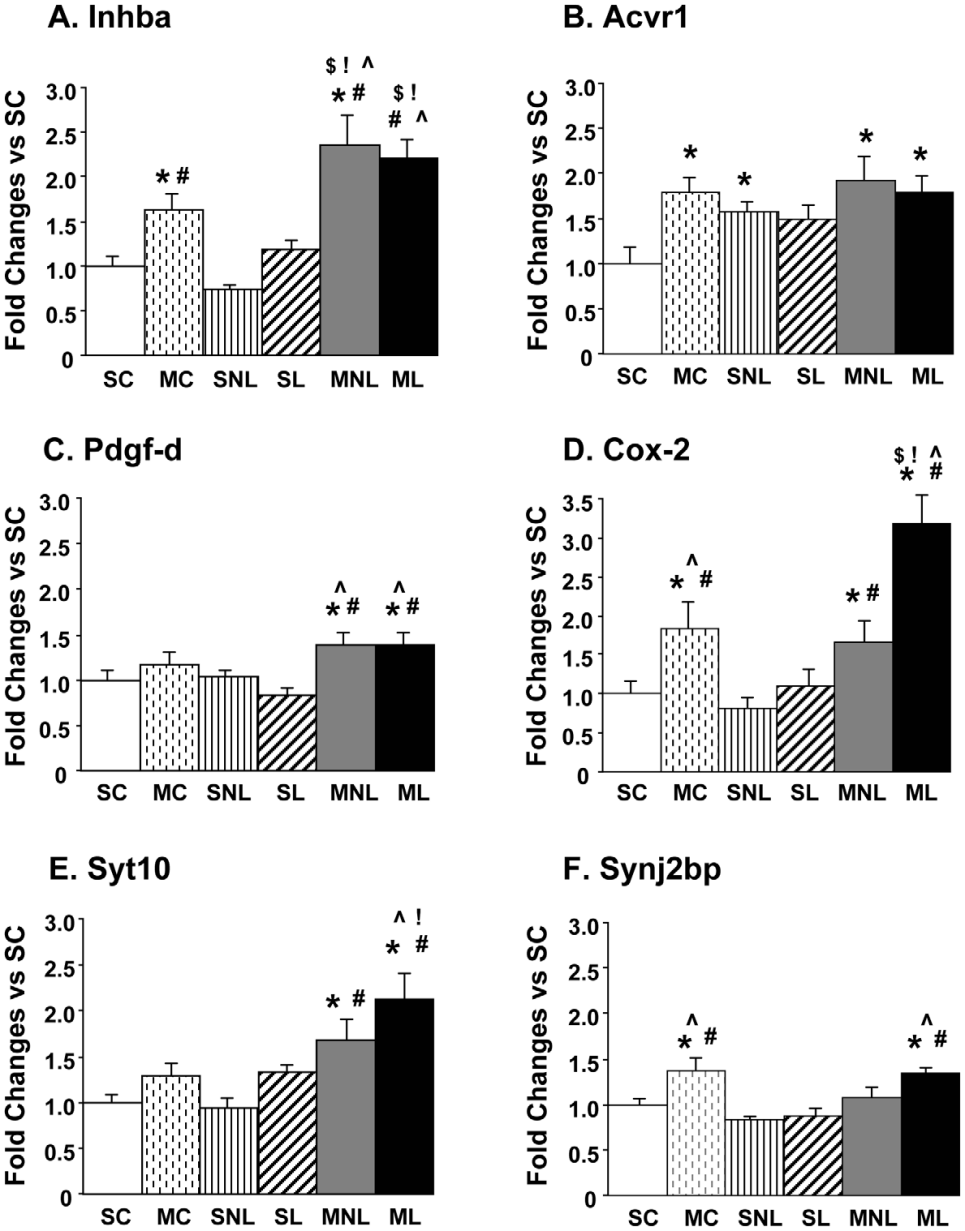
Effects of METH on the expression of *Inhba, Acvr1, Pdgf-d, Cox-2, Syt* and *Synj2bp* in DA-depleted striatum. The values represent means ± SEM (fold changes in comparison to the SC group) of 6–10 measurements. Statistical analyses were done by ANOVA followed by PLSD. Key to statistics: *p<0.05,in comparison to the SC group; #p<0.05, in comparison to the SNL group; !p<0.05, in comparison to the MC group; 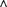
p<0.05, in comparison to the SL group; $p<0.05, in comparison to the MNL group.

Figure 4 illustrates the expression of some genes that showed METH-induced changes in the DA-depleted striatum. METH injection caused increases in *Inhba* in the intact (2.35-fold) (MNL) and denervated (2.21-fold) (ML) sides of the striatum in hemiparkinsonian rats (Figure 4A). METH injection also induced increases in *Inhba* in the control animals but these changes were of smaller magnitude than the increases in the lesioned animals (compare the MC to the MNL and ML groups). Figure 4B shows the effects of METH on the activin receptor, *Acvr1* [43]. Although not found on the array, we wanted to know if there were changes in this receptor in view of METH-induced changes in *Inhba*. We found that METH caused significant increases in *Acvr1* mRNA levels in the controls (MC, 1.79-fold), as well as on the intact (MNL, 1.93-fold) and lesioned (ML, 1.79-fold) sides of hemiparkinsonian rats. There were also significant increases (1.57-fold) in *Acvr1* expression in the intact side of saline-injected 6-OHDA-treated rats. The changes in the lesioned side of these animals did not reach statistical significance (1.49-fold, p = 0.06).

We also found significant METH-induced increases in *Pdgf-d* mRNA levels on both the intact (1.39-fold) and denervated (1.39-fold) striatum in comparison to the SC group and the intact (SNL) side of 6-OHDA-treated rats (Figure 4C). There were no significant changes in METH-treated control group (MC) and in 6-OHDA-treated animals injected with saline (SL). There were no significant effects of the 6-OHDA lesion on striatal *Cox-2* expression (Figure 4D). However, METH injection caused increases (MC, 1.83-fold) in the control group, as well as in the intact (MNL, 1.66-fold) and lesioned (ML, 3.19-fold) sides of the hemiparkinsonian rats. The METH-induced increases on the lesioned side (ML) were significantly higher than those observed in the MNL and MC groups (Figure 4D).

We found no significant changes in *Syt10* expression on the side of the lesion in animals treated with saline (Figure 4E). In addition, injection of METH caused no changes in striatum of controls animals (MC group). In contrast, the drug caused significant increases in *Syt10* expression in both the intact (1.68-fold) and DA-denervated (2.13-fold) sides of 6-OHDA-treated rats in comparison to the SC group and to the SNL side of hemiparkinsonian rats. Figure 4F shows that 6-OHDA did not cause any significant changes in the expression of *Synj2bp* in the saline-treated animals (SL). However, METH induced small but significant increases (1.35-fold) in *Synj2bp* in the DA depleted side (ML), but not on the intact side (MNL), of hemiparkinsonian rats. Unexpectedly, METH injection also caused small increases (1.35-fold) in *Synj2bp* in the control animals (MC group).

## Discussion

Injections of 6-OHDA in the MFB caused complete loss of DA and significant decreases in 5-HT content on the lesioned side of the striatum. The 6-OHDA lesion was also associated with METH-induced increases in 5-HIAA/5-HT ratios. 6-OHDA-induced striatal DA depletion resulted in substantial changes in transcript levels on the lesioned side. We also found that METH dose that caused changes in mRNA levels on the intact side elicited alterations in the expression of, mostly, different transcripts on the DA-depleted side. METH injection also failed to impact the expression of several genes whose mRNA levels were modulated by DA depletion in the saline-treated hemiparkinsonian rats.

### Effects of unilateral 6-OHDA-induced MFB lesions on striatal gene expression

We found significant increases in the expression of *Nts, Nmu,* and *Tac2* and decreases in *Tac1* expression in the DA-depleted striata of rats treated with saline. These results are consistent with previous reports of lesion-induced increases in *Nmu* [44], *Nts* [45], [46] and *Tac2* [47] as well as decreases in *Tac1* [18], [48]. Interestingly, although METH injection did not further influence the changes observed in *Tac1* and *Tac2* mRNA levels on the DA-depleted side, the drug did cause further increases in *Nmu* expression on the lesioned side, suggesting that *Nmu* expression is regulated by additional neurotransmitters in the absence of DA innervation. The observations that the small dose of METH caused significant increases in *Nts* mRNA levels in the control animals (MC) that were of lesser magnitude than those induced by the drug on the lesioned striatum suggest that METH might enhance *Nts* gene expression in association with or via increased 5-HT release, given that METH caused increased 5-HIAA/5-HT ratios on the 6-OHDA lesioned side. When taken together, our results suggest that the expression of *Tac2, Nmu,* and *Nts* might be under tonic inhibitory control by DA. The findings that this dose of METH did not cause any significant increases in *Tac2* and *Nmu* transcripts in the intact striatum suggest a potential DA-mediated floor effect under these conditions. The situation appears more complex for *Nts* mRNA which showed METH-mediated increases in both the presence and absence of DA. Increases in *Nts* mRNA after METH have been reported previously and are thought to be related to stimulation of DA D1 receptors [49]. However, the fact that METH caused greater increases in *Nts* transcript levels on the lesioned side suggests that both DA and 5-HT might be involved in regulating *Nts* transcript levels after injection of METH since methylenedioxymethamphetamine (MDMA) which causes 5-HT release can also increase *Nts* mRNA levels [50]. Other potential regulators of gene expression after 6-OHDA lesions might include changes in glutamatergic functions because hemiparkinsonian rats exhibit increases in extracellular glutamate [51]–[54] and changes in glutamate synapses [52] within the lesioned striatum. It is also possible to suggest the DA depletion-mediated changes in transcript levels might be related to alterations in the levels of *Nur77 (Nr4a1)* [55], the only transcription factor that showed 2-fold increases on the DA-denervated side (see Table 6). Therefore, there might exist a certain correspondence between DA depletion, *Nur77* expression, and up-regulated transcripts of some of the identified genes in the hemiparkinsonian brain.

It is also of interest to discuss the substantial increases in *Nmu* mRNA observed after DA depletion reported in the present study and in a previous microarray paper [44] because NMU is a neuropeptide which is found ubiquitously in the body, with very high levels reported in the gastrointestinal tract and the pituitary gland [56]. NMU was first isolated from porcine spinal cord [57]. Subsequently, NMU was located in the rodent brain and in other tissues [58]–[60]. Using radioimmunoassay, Domin et al. [59] reported high concentrations of NMU in the nucleus accumbens, septum, and hypothalamus but moderate levels in the substantia nigra and the globus pallidus and much lower levels in the striatum. Fujii et al. [61] used RT-PCR and reported that *Nmu* mRNA levels were very high in the pituitary. Other structures such as the medulla oblongata, hypothalamus and striatum showed moderate *Nmu* mRNA levels whereas the cerebral cortex, hippocampus, and the cerebellum had very low levels. NMU has also been reported to participate in several physiological functions including release of corticotrophin releasing hormone, excitation of nociceptive neurons, and regulation of body weight [62], [63]. NMU exerts its physiological effects by stimulation of the NMUR1 [61], [64] and NMUR2 [65] receptors. In the brain, the effects of this peptide might occur through NMUR2 because qPCR analysis failed to detect much *Nmur1* in the brain whereas moderate-to-high levels of *Nmur2* were found in the substantia nigra, the nucleus accumbens, and the striatum [66]. Because *Nmu* mRNA is expressed in the striatum and because its level is substantially regulated after DA depletion [44] and by METH administration (present study), it is possible to suggest that this neuropeptide might play important roles in striatal functions. This idea is supported by the report that intra-cerebroventricular NMU injections cause increased motor activity in rats [66].

The DA depletion-induced increases in *Fst* mRNA levels are consistent with those reported in a recent microarray study [44]. FST, a monomeric glycoprotein which is co-expressed with the activins, irreversibly binds with activins and prevents their interactions with their receptors [67]–[69]. Interestingly, we also found increases in *Inhba* transcript which encodes a subunit of inhibins and activins; both of which are members of a family of polypetides that also include transforming growth factor-beta and bone morphogenic proteins [70], [71]. Activins are disulfide-linked homodimers of INHBA (activin A) and of INHBB (activin B) and can form the heterodimer, activin AB [72]. Activin A is up-regulated in the brain consequent to kainate- [73] and ischemia- [74] induced insults. Because activins are also thought to have neurotrophic properties [75], the METH-induced increases in *Inhba* expression, and by extension of activins (or inhibins), suggest the possibility that this family of proteins might participate in METH-induced neuroplastic changes in the brain [37].

### Effects of METH injections on the expression of transcription factors in the rat striatum

Previous studies have also shown that the expression of several transcription factors is affected by injections of larger doses of METH in normal animals [29]–[31], [76]. These genes are similar to those reported by Berke et al. [16] who had used the DA D1 agonist, SKF38393, to report changes in gene expression mainly on the side of the 6-OHDA-induced DA depletion. In the present study, we used the indirect agonist, METH, which is dependent on DA release to exert its actions on DA receptors [21], [22], [24]–[26]. Interestingly, we found that the METH injection caused increases in *c-fos* mRNA levels on both sides of the hemiparkinsonian rats, with more prominent increases on the intact side (2.7-fold) than on the lesioned (1.75-fold) side (Table 6). The observations of increased *c-fos* mRNA levels on the lesioned side are consistent with previous reports that METH caused increase in c-Fos protein levels in the DA-depleted striatum [77]. Together, these observations suggest that METH-induced changes in *c-fos* mRNA levels might dependent on both DA and non-DA, presumably 5-HT, systems in the rodent striatum [78], [79]. The role of glutamate in these responses needs to be also considered since amphetamine-induced changes in striatal responses involves stimulation of NMDA receptors [80].

As reported above, we found that *Egr2* and *Egr3* transcripts were more induced by the METH injection on the intact side of the lesioned rats than in the control animals, whereas those in *Egr1* mRNA levels were similar in these two METH-treated groups. These results suggest that *Egr2* and *Egr3* transcription might be co-regulated in the rodent striatum in a manner comparable to observations in T cells [81]. The differential responses between the control animals and the hemiparkinsonian rats might be due, in part, to higher levels of baseline DA released in dialysates collected on the intact side of unilaterally 6-OHDA-lesioned rats in comparison to DA levels measured in control animals [24], [82]. Thus, the steady-state increases in DA released in the synaptic cleft of the 6-OHDA-treated animals might cause potentiated transcriptional sensitivity to METH-mediated DA-induced changes in *Egr2* and *Egr3* expression in the intact striatum.

### Differential effects of METH on gene expression between the DA-innervated and -depleted striatum of hemiparkinsonian rats

METH administration also caused substantial changes in the expression of several genes on the DA- depleted side of hemiparkinsonian rats (Figure 4). The *Pdgf-d* transcript is of interest because the PDGF-D protein belongs to a family of trophic factors that are involved in the growth and survival of mesenchymal cells [83], [84]. These factors include PDGF-A, PDGF-B, PDGF-C, and PDGF-D [85]. Although the effects of the PDGF-A and PDGF-B have been studied on diverse cell types of the nervous system [86], [87], little is known about the functions of PDGF-D in the brain. A previous study had found early and prolonged increases in neuronal *Pdgf-a* and *Pdgf-b* mRNA levels on the side ipsilateral to a unilateral 6-OHDA injection in the rat median forebrain bundle [88]. There were also more gradual increases in these transcripts on the contralateral side of the lesion [88]. Those findings differ from our present data because we found increases in *Pdgf-d* mRNA on both sides of the METH-treated hemiparkinsonian rats. Our observations that METH can cause increases in *Pdgf-d* mRNA levels on the lesioned side of hemiparkinsonian rats suggest that the PDGF-D protein might play a role in 5-HT-mediated changes after striatal DA depletion because of METH-induced increases in 5-HT turnover on the lesioned side. Because PDGF proteins act as proliferative factors for glial cells [89], [90], it will be of interest to examine to what extent PDGF-D might participate in METH-induced reactive astrocytosis or microgliosis [91]. Of related interest, we found that METH caused increases in the *Cox-2* transcript on both sides of the brains in hemiparkinsonian rats. These data are consistent with reports by other authors who have previously tabulated METH-induced changes in COX-2 protein expression [92], [93]. These METH-induced increases in COX-2 might be related, in part, to the increases in PDGF-D because *Cox-2* mRNA is induced during activation of microglial cells [94] and because PDGFs are proliferative factors for glial cells [89], [90].

Although we have focused the discussion on the known biochemical effects of METH on neurotransmitter release, it could also be argued that some of the changes in gene expression might be secondary to rotational behaviors, independent of drug effects. This is an important issue because immobilization of animals after apomorphine injection prevented priming responses to the direct DA D1 agonist, SKF38393, thus suggesting movement-mediated induction of these behavioral changes [95]. However, the fact that the pattern of changes in gene expression is similar in the striatum of the METH-treated control rats and the nonlesioned striatal side of the METH-treated hemiparkinsonian rats suggests that the changes in mRNA levels are secondary to drug effects not to intense turning behaviors since control rats exhibited no METH-induced rotation. The latter argument is consistent with the report that DA D1-induced behavioral responses are dissociated from changes in c-Fos protein expression [96]. This contention is also supported by the fact that administration of the indirect DA agonist, amphetamine, whose actions like those of METH [21]–[25] are also dependent on DA release from nerve terminals, did not cause priming to DA D1 receptor stimulation [96]. Nevertheless, the veracity of this claim will have to await results of time-dependent experiments in which animals are euthanized during the time of peak METH-induced rotation which occurred at around 30–45 min after the METH injection (see Figure 1B). The results of the proposed studies will be need to be contrasted to the present findings which were obtained in animals euthanized at 2 hours after the METH injection at a time when METH-induced rotation had subsided.

### Conclusion

In summary, we have reported that a single injection of a relatively low METH dose that caused substantial changes in gene expression in the intact striatum also triggered alterations in the expression of a different set of genes in the striatum that was completely lacking of DA. Because the 6-OHDA-induced lesion completely depleted dopamine in the striatum, these METH-induced changes on the lesioned are probably not related to disuse-induced supersensitivity of striatal DA receptors because METH is an indirect agonist that depends on intact DA terminals for its action on DA receptors. Thus, these observations implicate DA-independent phenomena in the METH-mediated regulation of these transcripts and point to the possibility that the absence of DA might cause plastic changes that render the striatum differentially responsive to the effects of METH on transcript levels in intrinsic striatal cells. This idea is consistent with the results of some studies that have documented differential physiological responses in the striatum of hemiparkinsonian rats [97]-[99]. It remains to be determined to what extent these changes might serve as substrates for synaptic plasticity observed in the lesioned striatum. Finally, our observations of METH-mediated DA-independent alterations in transcript levels suggest that other molecular pathways should be taken into consideration when discussing therapeutic approaches to METH abusers who show pathological changes, including DA depletion, in their brains.

## Supporting Information

Table S1 **List of rat primers used in quantitative PCR experiments.**
(PDF)

## References

[1] Cardoso F (2009). Huntington disease and other choreas.. Neurol Clin.

[2] Fahn S (2008). The history of dopamine and levodopa in the treatment of Parkinson's disease.. Mov Disord.

[3] Berger K, Przedborski S, Cadet JL (1991). Retrograde degeneration of nigrostriatal neurons induced by intrastriatal 6-hydroxydopamine injection in rats.. Brain Res Bull.

[4] Cadet JL, Zhu SM (1992). The intrastriatal 6-hydroxydopamine model of hemiparkinsonism: quantitative receptor autoradiographic evidence of correlation between circling behavior and presynaptic as well as postsynaptic nigrostriatal markers in the rat.. Brain Res.

[5] Cadet JL, Zhu SM, Angulo JA (1992). Quantitative in situ hybridization evidence for differential regulation of proenkephalin and dopamine D2 receptor mRNA levels in the rat striatum: effects of unilateral intrastriatal injections of 6-hydroxydopamine.. Brain Res Mol Brain Res.

[6] Herrera-Marschitz M, Arbuthnott G, Ungerstedt U (2010). The rotational model and microdialysis: Significance for dopamine signalling, clinical studies, and beyond.. Prog Neurobiol.

[7] Ungerstedt U (1971). Postsynaptic supersensitivity after 6-hydroxy-dopamine induced degeneration of the nigro-striatal dopamine system.. Acta Physiol Scand.

[8] Ungerstedt U, Arbuthnott GW (1970). Quantitative recording of rotational behavior in rats after 6-hydroxy-dopamine lesions of the nigrostriatal dopamine system.. Brain Res.

[9] Altar CA, Marien MR, Marshall JF (1987). Time course of adaptations in dopamine biosynthesis, metabolism, and release following nigrostriatal lesions: implications for behavioral recovery from brain injury.. J Neurochem.

[10] Angulo JA, Coirini H, Ledoux M, Schumacher M (1991). Regulation by dopaminergic neurotransmission of dopamine D2 mRNA and receptor levels in the striatum and nucleus accumbens of the rat.. Brain Res Mol Brain Res.

[11] Chritin M, Feuerstein C, Savasta M (1993). Time-course of changes in striatal levels of DA uptake sites, DA D2 receptor and preproenkephalin mRNAs after nigrostriatal dopaminergic denervation in the rat.. Brain Res Mol Brain Res.

[12] Graham WC, Crossman AR, Woodruff GN (1990). Autoradiographic studies in animal models of hemi-parkinsonism reveal dopamine D2 but not D1 receptor supersensitivity. I. 6-OHDA lesions of ascending mesencephalic dopaminergic pathways in the rat.. Brain Res.

[13] Angulo JA, Davis LG, Burkhart BA, Christoph GR (1986). Reduction of striatal dopaminergic neurotransmission elevates striatal proenkephalin mRNA.. Eur J Pharmacol.

[14] Campbell K, Wictorin K, Bjorklund A (1992). Differential regulation of neuropeptide mRNA expression in intrastriatal striatal transplants by host dopaminergic afferents.. Proc Natl Acad Sci U S A.

[15] Morris BJ, Herz A, Hollt V (1989). Localization of striatal opioid gene expression, and its modulation by the mesostriatal dopamine pathway: an in situ hybridization study.. J Mol Neurosci.

[16] Berke JD, Paletzki RF, Aronson GJ, Hyman SE, Gerfen CR (1998). A complex program of striatal gene expression induced by dopaminergic stimulation.. J Neurosci.

[17] El Atifi-Borel M, Buggia-Prevot V, Platet N, Benabid AL, Berger F (2009). De novo and long-term l-Dopa induce both common and distinct striatal gene profiles in the hemiparkinsonian rat.. Neurobiol Dis.

[18] Gerfen CR, Engber TM, Mahan LC, Susel Z, Chase TN (1990). D1 and D2 dopamine receptor-regulated gene expression of striatonigral and striatopallidal neurons.. Science.

[19] Konradi C, Westin JE, Carta M, Eaton ME, Kuter K (2004). Transcriptome analysis in a rat model of L-DOPA-induced dyskinesia.. Neurobiol Dis.

[20] Paul ML, Graybiel AM, David JC, Robertson HA (1992). D1-like and D2-like dopamine receptors synergistically activate rotation and c-fos expression in the dopamine-depleted striatum in a rat model of Parkinson's disease.. J Neurosci.

[21] Bustamante D, You ZB, Castel MN, Johansson S, Goiny M (2002). Effect of single and repeated methamphetamine treatment on neurotransmitter release in substantia nigra and neostriatum of the rat.. J Neurochem.

[22] Kuczenski R, Segal DS, Cho AK, Melega W (1995). Hippocampus norepinephrine, caudate dopamine and serotonin, and behavioral responses to the stereoisomers of amphetamine and methamphetamine.. J Neurosci.

[23] Stephans SE, Yamamoto BK (1994). Methamphetamine-induced neurotoxicity: roles for glutamate and dopamine efflux.. Synapse.

[24] Zetterstrom T, Herrera-Marschitz M, Ungerstedt U (1986). Simultaneous measurement of dopamine release and rotational behaviour in 6-hydroxydopamine denervated rats using intracerebral dialysis.. Brain Res.

[25] Zetterstrom T, Sharp T, Marsden CA, Ungerstedt U (1983). In vivo measurement of dopamine and its metabolites by intracerebral dialysis: changes after d-amphetamine.. J Neurochem.

[26] Shimada A, Yamaguchi K, Yanagita T (1996). Neurochemical analysis of the psychotoxicity of methamphetamine and cocaine by microdialysis in the rat brain.. Ann N Y Acad Sci.

[27] Abekawa T, Ohmori T, Koyama T (1994). Effects of repeated administration of a high dose of methamphetamine on dopamine and glutamate release in rat striatum and nucleus accumbens.. Brain Res.

[28] Nash JF, Yamamoto BK (1992). Methamphetamine neurotoxicity and striatal glutamate release: comparison to 3,4-methylenedioxymethamphetamine.. Brain Res.

[29] Beauvais G, Jayanthi S, McCoy MT, Ladenheim B, Cadet JL (2010). Differential effects of methamphetamine and SCH23390 on the expression of members of IEG families of transcription factors in the rat striatum.. Brain Res.

[30] Cadet JL, Jayanthi S, McCoy MT, Vawter M, Ladenheim B (2001). Temporal profiling of methamphetamine-induced changes in gene expression in the mouse brain: evidence from cDNA array.. Synapse.

[31] Jayanthi S, McCoy MT, Beauvais G, Ladenheim B, Gilmore K (2009). Methamphetamine induces dopamine D1 receptor-dependent endoplasmic reticulum stress-related molecular events in the rat striatum.. PLoS One.

[32] Wang JQ, McGinty JF (1995). Dose-dependent alteration in zif/268 and preprodynorphin mRNA expression induced by amphetamine or methamphetamine in rat forebrain.. J Pharmacol Exp Ther.

[33] Wang JQ, McGinty JF (1996). Acute methamphetamine-induced zif/268, preprodynorphin, and preproenkephalin mRNA expression in rat striatum depends on activation of NMDA and kainate/AMPA receptors.. Brain Res Bull.

[34] Li SJ, Jiang HK, Stachowiak MS, Hudson PM, Owyang V (1990). Influence of nigrostriatal dopaminergic tone on the biosynthesis of dynorphin and enkephalin in rat striatum.. Brain Res Mol Brain Res.

[35] Chritin M, Blanchard V, Raisman-Vozari R, Feuerstein C, Agid Y (1996). DA uptake sites, D1 and D2 receptors, D2 and preproenkephalin mRNAs and Fos immunoreactivity in rat striatal subregions after partial dopaminergic degeneration.. Eur J Neurosci.

[36] Krasnova IN, Betts ES, Dada A, Jefferson A, Ladenheim B (2007). Neonatal dopamine depletion induces changes in morphogenesis and gene expression in the developing cortex.. Neurotox Res.

[37] Krasnova IN, Justinova Z, Ladenheim B, Jayanthi S, McCoy MT (2010). Methamphetamine self-administration is associated with persistent biochemical alterations in striatal and cortical dopaminergic terminals in the rat.. PLoS One.

[38] Cadet JL, McCoy MT, Cai NS, Krasnova IN, Ladenheim B (2009). Methamphetamine preconditioning alters midbrain transcriptional responses to methamphetamine-induced injury in the rat striatum.. PLoS One.

[39] Krasnova IN, Li SM, Wood WH, McCoy MT, Prabhu VV (2008). Transcriptional responses to reinforcing effects of cocaine in the rat hippocampus and cortex.. Genes Brain Behav.

[40] Karstaedt PJ, Kerasidis H, Pincus JH, Meloni R, Graham J (1994). Unilateral destruction of dopamine pathways increases ipsilateral striatal serotonin turnover in rats.. Exp Neurol.

[41] Adams DH, Hanson GR, Keefe KA (2001). Differential effects of cocaine and methamphetamine on neurotensin/neuromedin N and preprotachykinin messenger RNA expression in unique regions of the striatum.. Neuroscience.

[42] O'Donovan KJ, Tourtellotte WG, Millbrandt J, Baraban JM (1999). The EGR family of transcription-regulatory factors: progress at the interface of molecular and systems neuroscience.. Trends Neurosci.

[43] Cameron VA, Nishimura E, Mathews LS, Lewis KA, Sawchenko PE (1994). Hybridization histochemical localization of activin receptor subtypes in rat brain, pituitary, ovary, and testis.. Endocrinology.

[44] Meurers BH, Dziewczapolski G, Shi T, Bittner A, Kamme F (2009). Dopamine depletion induces distinct compensatory gene expression changes in DARPP-32 signal transduction cascades of striatonigral and striatopallidal neurons.. J Neurosci.

[45] Bean AJ, During MJ, Deutch AY, Roth RH (1989). Effects of dopamine depletion on striatal neurotensin: biochemical and immunohistochemical studies.. J Neurosci.

[46] Hanson GR, Keefe KA (1999). Dopamine D-1 regulation of caudate neurotensin mRNA in the presence or absence of the nigrostriatal dopamine pathway.. Brain Res Mol Brain Res.

[47] Burgunder JM, Young WS (1989). Distribution, projection and dopaminergic regulation of the neurokinin B mRNA-containing neurons of the rat caudate-putamen.. Neuroscience.

[48] Gerfen CR, McGinty JF, Young WS (1991). Dopamine differentially regulates dynorphin, substance P, and enkephalin expression in striatal neurons: in situ hybridization histochemical analysis.. J Neurosci.

[49] Castel MN, Morino P, Dagerlind A, Hokfelt T (1994). Up-regulation of neurotensin mRNA in the rat striatum after acute methamphetamine treatment.. Eur J Neurosci.

[50] Adams DH, Hanson GR, Keefe KA (2005). 3,4-Methylenedioxymethamphetamine increases neuropeptide messenger RNA expression in rat striatum.. Brain Res Mol Brain Res.

[51] Garcia-Arencibia M, Ferraro L, Tanganelli S, Fernandez-Ruiz J (2008). Enhanced striatal glutamate release after the administration of rimonabant to 6-hydroxydopamine-lesioned rats.. Neurosci Lett.

[52] Meshul CK, Emre N, Nakamura CM, Allen C, Donohue MK (1999). Time-dependent changes in striatal glutamate synapses following a 6-hydroxydopamine lesion.. Neuroscience.

[53] Touchon JC, Holmer HK, Moore C, McKee BL, Frederickson J (2005). Apomorphine-induced alterations in striatal and substantia nigra pars reticulata glutamate following unilateral loss of striatal dopamine.. Exp Neurol.

[54] Yang J, Hu LF, Liu X, Zhou F, Ding JH (2006). Effects of iptakalim on extracellular glutamate and dopamine levels in the striatum of unilateral 6-hydroxydopamine-lesioned rats: a microdialysis study.. Life Sci.

[55] Paulsen RF, Granas K, Johnsen H, Rolseth V, Sterri S (1995). Three related brain nuclear receptors, NGFI-B, Nurr1, and NOR-1, as transcriptional activators.. J Mol Neurosci.

[56] Budhiraja S, Chugh A (2009). Neuromedin U: physiology, pharmacology and therapeutic potential.. Fundam Clin Pharmacol.

[57] Minamino N, Kangawa K, Matsuo H (1985). Neuromedin U-8 and U-25: novel uterus stimulating and hypertensive peptides identified in porcine spinal cord.. Biochem Biophys Res Commun.

[58] Conlon JM, Domin J, Thim L, DiMarzo V, Morris HR (1988). Primary structure of neuromedin U from the rat.. J Neurochem.

[59] Domin J, Ghatei MA, Chohan P, Bloom SR (1987). Neuromedin U—a study of its distribution in the rat.. Peptides.

[60] Minamino N, Kangawa K, Honzawa M, Matsuo H (1988). Isolation and structural determination of rat neuromedin U.. Biochem Biophys Res Commun.

[61] Fujii R, Hosoya M, Fukusumi S, Kawamata Y, Habata Y (2000). Identification of neuromedin U as the cognate ligand of the orphan G protein-coupled receptor FM-3.. J Biol Chem.

[62] Malendowicz LK, Nussdorfer GG, Markowska A, Tortorella C, Nowak M (1994). Effects of neuromedin U (NMU)-8 on the rat hypothalamo-pituitary-adrenal axis. Evidence of a direct effect of NMU-8 on the adrenal gland.. Neuropeptides.

[63] Peier A, Kosinski J, Cox-York K, Qian Y, Desai K (2009). The antiobesity effects of centrally administered neuromedin U and neuromedin S are mediated predominantly by the neuromedin U receptor 2 (NMUR2).. Endocrinology.

[64] Howard AD, Wang R, Pong SS, Mellin TN, Strack A (2000). Identification of receptors for neuromedin U and its role in feeding.. Nature.

[65] Shan L, Qiao X, Crona JH, Behan J, Wang S (2000). Identification of a novel neuromedin U receptor subtype expressed in the central nervous system.. J Biol Chem.

[66] Gartlon J, Szekeres P, Pullen M, Sarau HM, Aiyar N (2004). Localisation of NMU1R and NMU2R in human and rat central nervous system and effects of neuromedin-U following central administration in rats.. Psychopharmacology (Berl).

[67] Bilezikjian LM, Blount AL, Corrigan AZ, Leal A, Chen Y (2001). Actions of activins, inhibins and follistatins: implications in anterior pituitary function.. Clin Exp Pharmacol Physiol.

[68] Bilezikjian LM, Blount AL, Leal AM, Donaldson CJ, Fischer WH (2004). Autocrine/paracrine regulation of pituitary function by activin, inhibin and follistatin.. Mol Cell Endocrinol.

[69] Harrison CA, Gray PC, Vale WW, Robertson DM (2005). Antagonists of activin signaling: mechanisms and potential biological applications.. Trends Endocrinol Metab.

[70] Bernard DJ, Chapman SC, Woodruff TK (2001). Mechanisms of inhibin signal transduction.. Recent Prog Horm Res.

[71] Bilezikjian LM, Blount AL, Donaldson CJ, Vale WW (2006). Pituitary actions of ligands of the TGF-beta family: activins and inhibins.. Reproduction.

[72] Xia Y, Schneyer AL (2009). The biology of activin: recent advances in structure, regulation and function.. J Endocrinol.

[73] Tretter YP, Munz B, Hubner G, ten Bruggencate G, Werner S (1996). Strong induction of activin expression after hippocampal lesion.. Neuroreport.

[74] Bottner M, Dubal DB, Rau SW, Suzuki S, Wise PM (2006). Stroke injury in rats causes an increase in activin A gene expression which is unaffected by oestradiol treatment.. J Neuroendocrinol.

[75] Mukerji SS, Katsman EA, Wilber C, Haner NA, Selman WR (2007). Activin is a neuronal survival factor that is rapidly increased after transient cerebral ischemia and hypoxia in mice.. J Cereb Blood Flow Metab.

[76] Thomas DM, Francescutti-Verbeem DM, Liu X, Kuhn DM (2004). Identification of differentially regulated transcripts in mouse striatum following methamphetamine treatment—an oligonucleotide microarray approach.. J Neurochem.

[77] Ishida Y, Todaka K, Kuwahara I, Ishizuka Y, Hashiguchi H (1998). Methamphetamine induces fos expression in the striatum and the substantia nigra pars reticulata in a rat model of Parkinson's disease.. Brain Res.

[78] Gardier AM, Moratalla R, Cuellar B, Sacerdote M, Guibert B (2000). Interaction between the serotoninergic and dopaminergic systems in d-fenfluramine-induced activation of c-fos and jun B genes in rat striatal neurons.. J Neurochem.

[79] Rouillard C, Bovetto S, Gervais J, Richard D (1996). Fenfluramine-induced activation of the immediate-early gene c-fos in the striatum: possible interaction between serotonin and dopamine.. Brain Res Mol Brain Res.

[80] Konradi C, Leveque JC, Hyman SE (1996). Amphetamine and dopamine-induced immediate early gene expression in striatal neurons depends on postsynaptic NMDA receptors and calcium.. J Neurosci.

[81] Collins S, Lutz MA, Zarek PE, Anders RA, Kersh GJ (2008). Opposing regulation of T cell function by Egr-1/NAB2 and Egr-2/Egr-3.. Eur J Immunol.

[82] Zhang WQ, Tilson HA, Nanry KP, Hudson PM, Hong JS (1988). Increased dopamine release from striata of rats after unilateral nigrostriatal bundle damage.. Brain Res.

[83] Bergsten E, Uutela M, Li X, Pietras K, Ostman A (2001). PDGF-D is a specific, protease-activated ligand for the PDGF beta-receptor.. Nat Cell Biol.

[84] Heldin CH, Westermark B (1999). Mechanism of action and in vivo role of platelet-derived growth factor.. Physiol Rev.

[85] Andrae J, Gallini R, Betsholtz C (2008). Role of platelet-derived growth factors in physiology and medicine.. Genes Dev.

[86] Enge M, Wilhelmsson U, Abramsson A, Stakeberg J, Kuhn R (2003). Neuron-specific ablation of PDGF-B is compatible with normal central nervous system development and astroglial response to injury.. Neurochem Res.

[87] Woodruff RH, Fruttiger M, Richardson WD, Franklin RJ (2004). Platelet-derived growth factor regulates oligodendrocyte progenitor numbers in adult CNS and their response following CNS demyelination.. Mol Cell Neurosci.

[88] Funa K, Yamada N, Brodin G, Pietz K, Ahgren A (1996). Enhanced synthesis of platelet-derived growth factor following injury induced by 6-hydroxydopamine in rat brain.. Neuroscience.

[89] Dai C, Celestino JC, Okada Y, Louis DN, Fuller GN (2001). PDGF autocrine stimulation dedifferentiates cultured astrocytes and induces oligodendrogliomas and oligoastrocytomas from neural progenitors and astrocytes in vivo.. Genes Dev.

[90] Sjoborg M, Pietz K, Ahgren A, Yamada N, Lindvall O (1998). Expression of platelet-derived growth factor after intrastriatal ibotenic acid injury.. Exp Brain Res.

[91] Krasnova IN, Cadet JL (2009). Methamphetamine toxicity and messengers of death.. Brain Res Rev.

[92] Kita T, Shimada K, Mastunari Y, Wagner GC, Kubo K (2000). Methamphetamine-induced striatal dopamine neurotoxicity and cyclooxygenase-2 protein expression in BALB/c mice.. Neuropharmacology.

[93] Thomas DM, Kuhn DM (2005). Cyclooxygenase-2 is an obligatory factor in methamphetamine-induced neurotoxicity.. J Pharmacol Exp Ther.

[94] Thomas DM, Francescutti-Verbeem DM, Kuhn DM (2006). Gene expression profile of activated microglia under conditions associated with dopamine neuronal damage.. Faseb J.

[95] Simola N, Di Chiara G, Daniels WM, Schallert T, Morelli M (2009). Priming of rotational behavior by a dopamine receptor agonist in Hemiparkinsonian rats: movement-dependent induction.. Neuroscience.

[96] Paul ML, Currie RW, Robertson HA (1995). Priming of a D1 dopamine receptor behavioural response is dissociated from striatal immediate-early gene activity.. Neuroscience.

[97] Azdad K, Chavez M, Don Bischop P, Wetzelaer P, Marescau B (2009). Homeostatic plasticity of striatal neurons intrinsic excitability following dopamine depletion.. PLoS One.

[98] Centonze D, Gubellini P, Rossi S, Picconi B, Pisani A (2005). Subthalamic nucleus lesion reverses motor abnormalities and striatal glutamatergic overactivity in experimental parkinsonism.. Neuroscience.

[99] Day M, Wang Z, Ding J, An X, Ingham CA (2006). Selective elimination of glutamatergic synapses on striatopallidal neurons in Parkinson disease models.. Nat Neurosci.

