# Supplementary material for: Methamphetamine-Induced Dopamine-Independent Alterations in Striatal Gene Expression in the 6-Hydroxydopamine Hemiparkinsonian Rats
Source: PLoS One. 2010 Dec 13;5(12):e15643. doi: 10.1371/journal.pone.0015643 (PMC3001483; doi:10.1371/journal.pone.0015643)
Supplement: Table S1 — List of rat primers used in quantitative PCR experiments. (PDF) [file pone.0015643.s001.pdf]

Table S1. List of rat primers used in quantitative PCR experiments

Gene Name	Forward	Reverse
Inhba	GCT TCA TGT GGG TAA AGT	CTG CCT TCC TTG GAA ATC
Acvr1	TGG TGA GCA ATG GTA TAG TG	CAG AGA AGT TAA TGT CGG G
Pdgf-d	AAG ATG GTG TGG CCA TA	AGT TGA TCT CTG AGG CT
Cox-2	CCG GGT CTG ATG ATG TA	CTC AGG TGT TGC ACG TA
Syt10	CGA CCG ATC CTA CAT CT	GAG TCA ACT GAT TTC TGC TT
Synj2bp	AGT GCA GAA TGG ACC TA	GAC ACA CTG GAA GAC AAC
Tac1	GGC ATG GTC AGA TCT CTC A	TGA ATA GAT AGT GCG TTA CAG G
Tac2	CCC TGG ACT GCA TCA TAA A	GGA CAC TAC AGA AAC CAT AAT
Pdyn	CTT CAT CCT CCT CTG CTT A	CGG ACA CTG GAT GGA TT
Nts	TTG CAG CCT GAT CAA TAA C	TCC TGA ATT ATC TCC CAG TG
Nmu	CCA CCA GAA CAA GAA CTA C	GAC AGA CGA CAC AAC AT
Fst	CCC TTG TAA AGA AAC GTG	CAC ATT CGT TGC GGT AG
Clathrin, light chain	AAG TAT CCG TAA GTG GAG	GGG GTT AAA GTC ACA CAG
